# Abnormal Pregnancy Outcomes in Mice Using an Induced Periodontitis Model and the Haematogenous Migration of *Fusobacterium nucleatum* Sub-Species to the Murine Placenta

**DOI:** 10.1371/journal.pone.0120050

**Published:** 2015-03-25

**Authors:** Sara Stockham, Justine E. Stamford, Claire T. Roberts, Tracy R. Fitzsimmons, Ceilidh Marchant, P. Mark Bartold, Peter S. Zilm

**Affiliations:** 1 Oral Microbiology, School of Dentistry, The University of Adelaide, Adelaide, Australia; 2 Robinson Research Institute, The University of Adelaide, Adelaide, South Australia, Australia; 3 Colgate Australian Clinical Dental Research Centre, School of Dentistry, The University of Adelaide, Adelaide, Australia; Xavier Bichat Medical School, INSERM-CNRS—Université Paris Diderot, FRANCE

## Abstract

**Objectives:**

To investigate if there is subspecies specific migration to the placenta by *Fusobacterium nucleatum* (*Fn*) and to determine whether experimentally induced periodontitis results in adverse pregnancy outcomes (APO) in mice.

**Methods:**

Periodontitis was induced in pregnant mice using an inoculum of *Fn* and *Porphyromonas gingivalis*. In parallel, four sub-species of *Fn* were individually injected into the circulatory system. At day 18 of gestation, the placenta, liver, spleen and blood were harvested and litter size, number of viable fetuses and resorptions, maternal, fetal and placenta weights were recorded. For the direct inoculation group, some mice were allowed to deliver for assessment of length of gestation, litter size, maternal, placental and pup weight. The presence of *Fn* was assessed by PCR and inflammatory mediators were measured by ELISA or multiplex analysis.

**Results:**

Mice with alveolar bone loss, a marker of periodontitis, demonstrated significantly higher fetal weights (p = 0.015) and fetal/placental weight ratios (p = 0.030). PCR analysis of maternal organs did not identify *Fn* in any extracted tissues. In mice that received direct injection of *Fn* subspecies, varying degrees of APO were observed including preterm birth, intrauterine growth restriction, and fetal loss. Haematogenous spread of only *Fn* subsp. *nucleatum* to the placenta was confirmed. Litter size was significantly smaller (p = 0.023) and the number of resorptions was higher in inoculated versus control groups. Mice injected with subsp. *nucleatum* had significantly increased circulating CRP levels (p = 0.020) compared to controls while the mice with induced periodontitis had increased levels of IL-6 (p = 0.047) and IL-8 (p = 0.105).

**Conclusions:**

Periodontitis in mice elevated fetal weight and the fetal weight/placental weight ratio. This study found that subsp. *nucleatum* migrated haematogenously to the placenta, leading to APO in mice. The study supports the potential role of *Fn* in the association between periodontitis and APO.

## Introduction

Periodontal disease is the result of complex interactions between the host and certain microbial species residing in the sub-gingival environment. It is an inflammatory disease where bacteria and their products induce an inflammatory response within the periodontal tissues [1]. Although the host response is primarily protective, in conjunction with bacterial virulence factors it can result in the progressive destruction of the periodontal ligament, gingival recession, loss of alveolar bone, tooth mobility and ultimately tooth loss. It is a serious disease which potentially allows for oral bacteria to disseminate around the body and cause major complications to human health including adverse pregnancy outcomes (APO) [2,3].

A recent review proposed that infection or challenge to the feto-placental unit by oral pathogens is the most important biological pathway leading to APO as it is thought that intrauterine infection may account for 25–40% of pre-term births (PTB) [4]. The hormonal changes associated with pregnancy predispose women to periodontal disease and gingival bleeding allows for the continual release of bacteria or their by-products into the circulatory system. Increased levels of oestrogen and progesterone also increase the vascular permeability of the periodontium facilitating transmission to the feto-placental unit [4].

Many epidemiological and intervention studies have been undertaken to assess the relationship between periodontal disease and APO. However, methodological inconsistencies, population differences, relative obstetric risk and sample size have led to equivocal results [4–14]. It has been reported that the prevalence of periodontitis in the Australian female population is 19.0% [15] and as part of the US National Health and Nutrition Examination Survey cycle, 64.7 million adults (47% of the sample) had periodontitis distributed as 8.7% mild, 30% moderate and 8.5% severe [16]. APO in humans are also a significant health concern, The World Health Organisation in 2005 estimated that 12.9 million PTBs occurred world-wide and 42% of these resulted in perinatal mortality [17]. Pregnancy complications can lead to respiratory, cardiovascular and neurological disorders including auditory and ophthalmic impairments as well as cognitive and learning disabilities and cerebral palsy that could affect the infant throughout life [18]. The impact of periodontitis on human health is therefore potentially far greater than its primary disease outcome.

APO caused by the haematogenous transfer of oral bacteria to the feto-placental unit challenges the predominant paradigm that infections originate from the lower genital tract and ascend to the sterile fetal membranes and placenta [19,20]. The hypothetical mechanistic model [21] proposes that APO could result from either the direct transit of oral bacteria or their pathogenic products to the feto-placental unit leading to infection and /or a localised inflammatory response. Alternatively APO can result from indirect pathways whereby inflammatory cytokines or acute phase reactants produced in the gingival tissue or liver, respectively, are released into the circulation and reach the feto-placental unit causing an imbalance in the strictly regulated innate pro-inflammatory immune status within the uterus [4,21,22]. More recently it has been proposed that the placenta harbors its own microbiome and the colonisation of the placenta by oral commensals may be facilitated by the immune suppression in the placenta during pregnancy [20,23]. APO may occur as a result of a heightened inflammatory response caused by the dysbiosis of the placental microbiome that may tip the balance in favour of fetal rejection [20]. Inflammation can also lead to other pregnancy complications, elevated levels of the pro-inflammatory cytokines Interleukin-6 (IL-6) and Tumour Necrosis Factor—alpha (TNF-α) for example interfere with insulin signalling and lead to glucose intolerance resulting in gestational diabetes mellitus [4].

Investigation of the potential link between periodontitis and APO in many animal studies has implicated certain microbial species which are among the 700 taxa which reside within the oral milieu. During development of the periodontal biofilm species associated by the so-called orange cluster of increase in number and are thought to induce environment al changes conducive to the colonisation of the more pathogenic species associated with the red cluster [24]. The ability to alter environmental conditions and invade human cells may be an important factor which allows the orange cluster organisms to disseminate and grow in other biological niches [25,26]. *Fusobacterium nucleatum*, a Gram-negative anaerobe, is ubiquitous in the oral cavity and increases in number in periodontal disease [27]. *F*. *nucleatum* also forms aggregates with other bacteria and acts as a bridge between colonisation of early and late bacteria during the development of the sub-gingival biofilm [28].

*F*. *nucleatum*, is the most frequently isolated bacterial species in APO including PTB, stillbirth, and early-onset neonatal sepsis [2]. It is infrequently isolated from the vagina so its detection in amniotic fluid, fetal membranes, cord blood, neonatal gastric aspirates, fetal lung and stomach suggests that the organism is capable of causing infection in sites distant from the oral cavity and has led to its identification as an emerging pathogen of medical significance [29–31]. More recent studies have also suggested that haematogenous migration of *F*. *nucleatum* may be linked to heart disease, rheumatoid arthritis, colorectal cancer and inflammatory bowel disease [32–35].

The reason why *F*. *nucleatum* is potentially capable of translocation to the feto-placental unit is in part due to its metabolic diversity and ability to invade endothelial and epithelial cells [36–39]. *F*. *nucleatum* also stimulates a toll-like receptor 4-mediated inflammatory response that in itself is able to affect birth outcomes [25,40]. Genetically, the species is extremely heterogeneous. Five subspecies have been identified; *nucleatum*, (*FNN*) *polymorphum*, (*FNP*) *vincentii*, (*FNV*) and *fusiforme* (*FNF*) and *animalis* (*FNA*) [41,42]. It is suggested that different subspecies may vary in their pathogenesis and be related to different levels of disease activity [43]. Genomic comparisons have shown that 919 genes account for the differences between *FNP*, *FNN*, and *FNV* [44,45]. *FNP* represents a separate phylogenetic branch which also includes significant human pathogens [46].

The accumulating evidence suggests a plausible biological association between periodontal disease and APO’s. However, evidence showing the causal link between APO and periodontal disease in mice has, to date, relied on a short-term bacteraemia caused by the injection of oral bacteria intra-venously (I.V.) into mice or by mimicking a chronic infection by injecting oral bacteria into a cylinder placed subcutaneously [19,27,47]. Arce *et al*., (2009) used an oral infection model of periodontitis in pregnant mice to examine the effect on fetal growth, fecundity and expression of TLR4 following an axenic or mixed inoculum containing *Campylobacter rectus* [25]. Evidence of alveolar bone loss typical of a chronic inflammatory response associated with periodontal disease was not performed however.

In the present study, we have experimentally induced periodontitis in pregnant mice to determine if *F*. *nucleatum* and/or *Porphyromonas gingivalis* are able to migrate from the periodontium to the placenta and/or induce APO. In parallel we also followed published protocols whereby four different *F*. *nucleatum* subsp. were injected I.V. into pregnant mice.

The first aim was to determine if the haematogenous migration of *F*. *nucleatum* to the murine placenta and its effect on APO was strain specific. The second aim of the study was to use a murine oral gavage model of periodontitis to determine if APO could be significantly increased as a result of inducing periodontitis. Thirdly, we aimed to compare the key immunological changes in pregnant mice following induced periodontitis or I.V. injection of different *F*. *nucleatum* subsp. and relate these to observed APO. The results overall support the contention that periodontal disease increases maternal inflammatory activity potentially causing APO.

## Materials and Methods

This investigation was approved by the University of Adelaide Animal Ethics Committee (ethics approval number M-2011-153 and M-2011-130).

### *F*. *nucleatum* type strains

Subspecies, *nucleatum* (ATCC 25586 isolated from a cervico-facial lesion), *vincentii* (ATCC 49256 isolated from human periodontal pocket), *polymorphum* (ATCC 10953 isolated from human inflamed gingiva) and *fusiforme* (ATCC 11326 isolated from sinusitis in upper jaw) were purchased from the ATCC (Cryosite, NSW, Australia) and were maintained on anaerobic blood agar (Oxoid, Vic. Australia) under an atmosphere of carbon dioxide, hydrogen and nitrogen mixed as a ratio of 5:5:90.

For the tail vein inoculation of mice, broth cultures were grown anaerobically overnight at 37°C in Brain-Heart Infusion broth (Oxoid). Contamination was periodically monitored by Gram-staining and plating onto anaerobic blood agar containing vancomycin (Oxoid).

### I.V. injection experiments

8–10 week old female BALB/c mice were mated at a ratio of 1female:1male. Mating was indicated by the presence of a copulatory plug, and designated as day one of gestation. Fourteen mice were inoculated via tail vein injection with an inoculum (100μL) of only one of each *F*. *nucleatum* subspecies (suspended in 0.9% w/v NaCl) at day 16 of their 20–21 day gestation. This time was chosen to mimic late gestation in women, (28–32 weeks). The inoculum was adjusted using a NanoDrop 2000c spectrophotometer (Thermo Scientific, Waltham, MA. USA) to give an optical density of 1.0. 100μL was injected into the tail vein which was approximately 0.5% of the total body weight of the mouse. A serial dilution of the inoculum was performed to determine the number of viable bacteria which was consistently in the range of 10 ^7^–10 ^8^ cfu/ml. Control mice were injected with 100μL saline and two mice were injected with 100μL heat killed *FNN* suspended in saline. Heat killed cultures were prepared by placing the inoculum at 50°C for 30min. Aliquots were then plated onto anaerobic blood agar and incubated anaerobically to check for viability.

At day 18 of gestation, 3 pregnant mice from each group were sedated using a chamber containing isoflurane so that blood could be collected by cardiac puncture before being humanely euthanased and dissected to harvest samples of liver, spleen, all fetuses and placentas. Blood was placed immediately into a heparin tube and liver, spleen and placenta were immediately placed into liquid nitrogen and stored until analysis of genomic DNA by Polymerase Chain Reaction (PCR). The remaining 11 mice from each group were left to deliver to assess length of gestation, litter size, maternal and pup weight. Mice were checked daily after injection for signs of ill health or delivery and any pups born were weighed within 4–6 hours following delivery.

Intrauterine growth restriction (IUGR) and low birth weight (LBW) were defined as fetus or pup weight respectively being 2 standard deviations smaller than average weight of the controls.

### Murine Periodontitis model

The murine model of periodontitis used in the present study was first described by Baker *et al*. (2000) and subsequently modified by Bendyk *et al*. [48,49]. Following treatment with Kanamycin (1mg/mL) twenty five BALB/c mice (5–7 weeks old) were inoculated over four weeks with either an inoculum containing each of the four subsp. (*FNN*, *FNP*, *FNF*, *FNV*) and *Porphyromonas gingivalis* (W50) suspended in 2% (v/v) carboxymethyl cellulose (CMC) or CMC only as previously described [49]. Mice were caged with soft, sterile bedding free of antibiotics and fed powdered, sterile, non-granular food to prevent impaction of food around the gingiva. Female mice were then placed with males at a ratio of 1:1 for 3 days or until the identification of a vaginal plug which was recorded as day 1 of gestation. During gestation, oral inoculations of bacteria or CMC only were maintained twice weekly. At day 18 of gestation, all pregnant mice were sedated so that blood could be collected before being humanely euthanased and dissected to harvest samples of placenta, liver, spleen as previously described. In addition, weights of the individual fetuses and placentas (with the endometrium removed) were also recorded.

The mandibles and maxillae of all mice were harvested by sharp dissection at the same time as tissue dissection. The jaws were fixed in 10% formalin for a minimum of 48 hours before being defleshed both mechanically and via the use of 1% sodium hydroxide solution (NaOH). The jaw bones were washed in saline and dried at 40°C.

### Measurement of alveolar bone loss

Alveolar bone loss was assessed with digital imaging on a Leica MZ16FA stereo microscope with a magnification of 32X (Leica Microsystems, Wetzlar, Germany). The dried maxillae and mandibles were stained with 1% aqueous methylene blue to highlight the cemento-enamel junction (CEJ) and crest of the alveolar bone. They were mounted on a rotatable and lockable stage that allowed all samples to be positioned identically under the microscope for comparison between groups and between specimens. Bone loss was identified visually by an increased distance between the CEJ and alveolar bone crest of each sample as previously described [49]. The exact quantitative assessment of bone loss was unable to be determined as the area originally occupied by the biologic width (the connective tissue and epithelium that is located within the CEJ and alveolar crest) is unknown, and therefore mice with alveolar bone loss greater than 0.12mm^2^ per tooth were only included in the experimental group.

### Polymerase Chain Reaction

DNA isolation from tissues and blood were completed using the QIAamp DNA Mini Kit (Qiagen, Limberg, Netherlands) as per manufacturer’s instructions with blood (200 μL), liver (25mg), spleen (10mg) and placenta (50mg) to yield optimal DNA from isolation. A sample of the placenta from the saline control group was “spiked” with *FNN* as a positive control. The concentration and quality of the nucleic acid was obtained for each purified sample using the NanoDrop 2000c spectrophotometer based on the 260/280nm ratio [50].

PCR was initially performed on liver, spleen and blood samples. DNA extracted from tissue samples was diluted to 10ng/μL. As a positive control, DNA extracted from each subspecies were diluted to 1ng/μL. PCR was performed in a total volume of 25μl in strip PCR tubes (Axygen, New York, NY, USA). ThermoPol Buffer, dNTPs and Taq polymerase were purchased from New England BioLabs (MA, USA) and primers were purchased from Geneworks (Adelaide SA, Australia). PCR reaction mixtures contained, 1X ThermoPol buffer, 200μM dNTP’s, 400nM forward primer, 400nM reverse primer, 0.625 units Taq polymerase and 10ng genomic DNA. To check for DNA contamination, every PCR run included a control which did not contain genomic DNA (NTC-no template control). Mouse genomic DNA isolated from the placenta from a control mouse was also included as a negative control.

Visualisation of PCR products was performed on a 2% agarose gel in 0.5X TBE buffer. Wells were loaded with 5μL of a mixture containing 1.0μl 6X loading dye (New England BioLabs) 1.0μL SYBR green (diluted 1:200, Life Technologies, NY. USA) and 5μL PCR reaction mixture. Molecular size was estimated by comparison to the 2-Log DNA ladder standard (New England BioLabs). PCR products were visualised by scanning with a Typhoon TRIO^+^fluorescence scanner (GE Healthcare, CA, USA).

The 16S rRNA gene was used initially to screen for the presence of *F*. *nucleatum* DNA. The amplification reactions were performed in an automated thermal cycler (T100 Thermocycler, BioRad, CA, USA), programmed for denaturation at 95°C, for 3 minutes, followed by 34 cycles of denaturation at 95°C for 10 seconds, annealing at 48–60°C, for 30 seconds and extension at 68°C, for 30 seconds and a final extension at 68°C for 3 minutes. If the samples returned a positive result, primers specific for each sub-species [48] were substituted to confirm the presence of the subsp. used in the inoculum (Table 1). For the detection of *P*. *gingivalis*, DNA specific primers were used (Table 1) and PCR conditions remained the same as those used for *F*. *nucleatum*.

**Table 1:** Primer sequences used to identify the presence of all or individual *F*. *nucleatum* subspecies and *P*. *gingivalis*.

*F*. *nucleatum* subspecies	Primer sequence (5’–3’)	Amplicon length
FNN	FN-SL-F tggttggttcggtaagttc	383bp
	FN-SL-R-nuc cgtatttcccttagcctcatttg	
FNF	Fs17-F14 gatgaggatgaaaagaaacaaagta	393bp
	Fs17-R14 ccattgagaagggctattgac	
FNP	FN-SL-F tggttggttcggtaagttc	388bp
	FN-SL-R-2poly tcatttgtatttcctttagcttg	
FNV	Fv35-R1 ataatgtgggtgaaataa	208bp
	Fv35-F1 cccaaggaaaatactaa	
All subspecies	16SrRNA-27F agagtttgatcctggctcag	∼400bp
	FNUC-16SrRNA-R gtcatcgtgcacacagaattgctg	
*P*. *gingivalis* (W50)	PG16S-F aggcagcttgccatactgcg	404bp

FNN, subsp. nucleatum; FNF, subsp. fusiforme; FNP, subsp. polymorphum; FNV, subsp. vincentii.

### Measurement of Inflammatory Mediators in Serum

Serum collected at day 18 of pregnancy was analysed for the presence of IFNγ, IL-1β, IL-6, IL-10, TNF-α together with mouse homologues of IL-8 (KC, LIX, MIP-2) using a multiplex assay according to the manufacturer’s protocol (Milliplex MAP kit, Merck Millipore, Billerica, MA, USA). All standards and samples were assayed in duplicate using a Luminex 200™ System (Luminex Corporation, Austin, TX, USA). Concentrations were determined from standard curves and analysed using xPONENT version 3.1 software (Luminex Corporation).

Mouse C-Reactive Protein was measured in duplicate using a commercially available Enzyme Linked Immunosorbent Assay (R&D Systems, Minneapolis, MN. USA) and optical density (450nm) read on a Powerwave microplate reader (BioTek Instruments, Winooski, VT, USA). Standard curves were generated using KC4 software (BioTek Instruments) and used to determine the concentration of CRP in each sample.

### Statistical analysis

The data were analysed using IBM SPSS Version 17. Fetal weight to placenta weight ratios were calculated. Linear mixed model ANOVA was used to assess the effect of inoculation on placenta, fetus and pup data and these were adjusted for the number of viable fetuses and litter size. A multivariate general linear model was used to analyse maternal data including maternal weight, resorptions, stillbirths, gestational age, litter size, and fetal viability. Multiple comparisons were assessed using SIDAK Post Hoc test.

## Results

### I.V. injection of *F*. *nucleatum* subspecies

**Fig 1:**
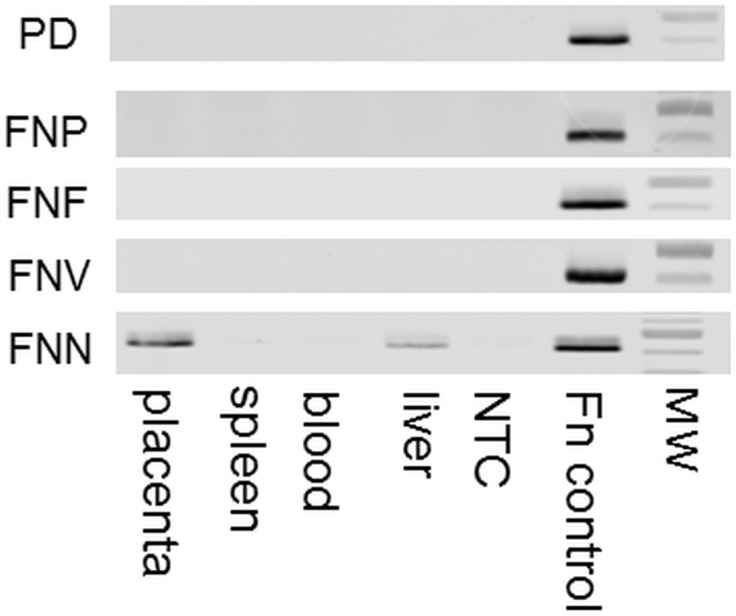
Detection of *F*. *nucleatum* in mouse placenta, blood, spleen and liver by PCR. Detection of *F*. *nucleatum* after experimentally induced periodontal disease (PD) with all subspecies or I.V. injection of subsp. *nucleatum* (FNN). *polymorphum* (FNP) *fusiforme* (FNF) and *vincentii* (FNV). Molecular weight (MW) markers shown represent 500bp (darker band) and 400bp. NTC is no template control.

When mice were inoculated with a single subspecies, only *FNN* was detected in the placenta of 80% of mice injected with this subspecies (Fig. 1; Table 2). This was also the only subspecies to induce fetal miscarriage (resorption) 48 hours after injection (Table 2). *FNF*, *FNP* and *FNV* did not show evidence of placental colonisation, (Fig. 1) although mice inoculated with *FNP* and *FNV* exhibited adverse pregnancy outcomes such as LBW, stillbirth and PTB, (Table 2). None of the *F*. *nucleatum* subsp. were detected in the blood or spleen of any mouse although a band in one of the *FNN* liver samples was present (Fig. 1) suggesting a systemic infection. When mice were inoculated with heat killed *FNN*, the placenta, liver, spleen and blood samples were also negative and mice had no observed APOs.

**Table 2:** Summary of adverse pregnancy outcomes in mice following tail vein injection with *F*. *nucleatum* subspecies.

Group	Placental colonisation	Fetal resorption	Low birth weight	Preterm birth	Stillbirth
Control	N/A	No	No	No	No
FNN	Yes 80%	Yes 40%	Yes	No	No
FNP	No	No	Yes	No	Yes 11%
FNF	No	No	No	No	No
FNV	No	No	Yes	Yes 87.5%	Yes 0.05%

FNN, subsp. nucleatum; FNF, subsp. fusiforme; FNP, subsp. polymorphum; FNV, subsp. vincentii.

**Fig 2:**
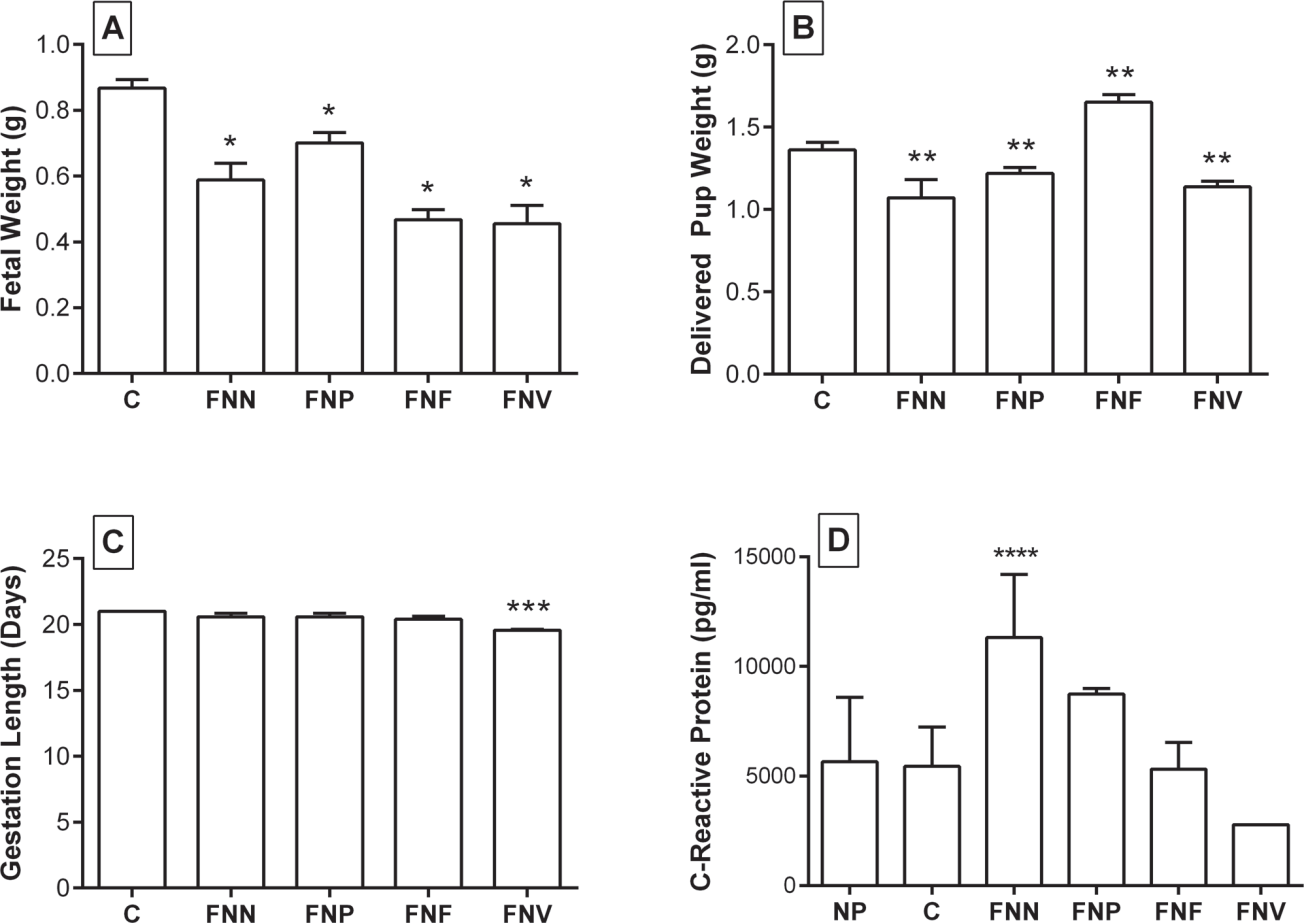
Birth outcomes and CRP levels in pregnant mice following I.V injection of *F*. *nucleatum*. Estimated marginal mean of (A) fetal weight at day 18; (B) delivered pup weight; (C) length of gestation of all mice; (D) C-Reactive protein concentration in serum of pregnant mice after inoculation with *F*. *nucleatum* subsp. *nucleatum* (FNN), *polymorphum* (FNP), *fusiforme* (FNF), *vincentii* (FNV). Non-pregnant (NP), Saline control (C). Bars represent the mean ± SEM. All statistically significant differences were in comparison to saline control *P ≤ 0.001; **P ≤ 0.02; ***P = 0.005; ****P = 0.020.

When adjusted for litter size, in all mice challenged with *F*. *nucleatum*, fetal weight at day 18 (Fig. 2A) and pup weight (Fig. 2B), were significantly different when compared with the control group. Maternal inoculation with *FNV*, *FNN* and *FNP* resulted in smaller fetuses and pups compared to control mice. Inoculation with *FNF* resulted in newborn pups that were significantly heavier compared to controls (p < 0.001). Interestingly, *FNF* inoculation significantly reduced placental weight, (0.089 ± 0.004g versus 0.114 ± 0.004g, p < 0.001) with no difference in litter size.

All mice receiving tail vein injection of *FNN*, *FNP and FNF* did not show a significant decrease in gestation time compared with the control group (Fig. 2C). In the group inoculated with *FNV*, significant differences (p = 0.005) were observed as over 87% of pups were delivered prematurely (Table 2).

Stillborn pups accounted for 11% of all pups born to mice challenged with *FNP*, and 0.05% of pups born to mice challenged with *FNV* (Table 2). Neither were significantly different from controls. Mean maternal weights at day 18, were not different between groups (p = 0.119).

Despite low pregnancy rates (50–66%) affecting the sample size, at day 18 of gestation, mice injected with *FNN* but not other species, showed a significant (p = 0.020) increase in CRP serum concentration compared to the pregnant control mice (Fig. 2D). There was also no significant difference in CRP levels between pregnant and non-pregnant females.

### Induced periodontitis model

Fifteen of the twenty-five experimental mice and ten of the twenty control mice became pregnant. Only pregnant mice were used for subsequent analyses. *F*. *nucleatum* or *P*. *gingivalis* was not detected by PCR in any control or experimental liver, spleen or placental samples.

**Fig 3:**
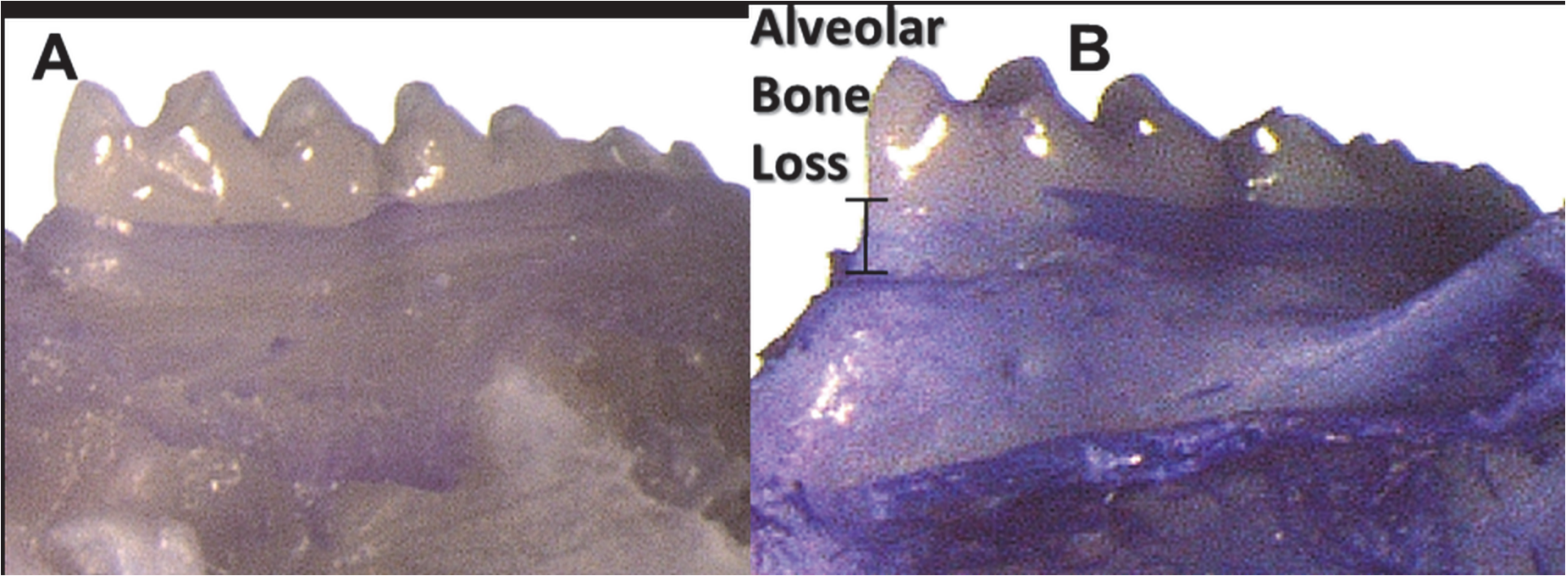
Experimentally induced alveolar bone loss. Buccal view of mandibular left molars in (A) control mouse (CMC inoculations); (B) experimental mouse (*P*. *gingivalis* and *F*. *nucleatum* inoculations). Observable alveolar bone loss in (B) compared with (A) as indicated by the distance from the cemento enamel junction (CEJ) and alveolar crest.

Alveolar bone loss as detected by increased distance from the CEJ to the alveolar crest in either the maxilla or the mandible in each mouse was evident in 12 of the 15 pregnant experimental mice and in none of the 10 pregnant control (mice that were inoculated with CMC only) mice (Fig. 3).

**Fig 4:**
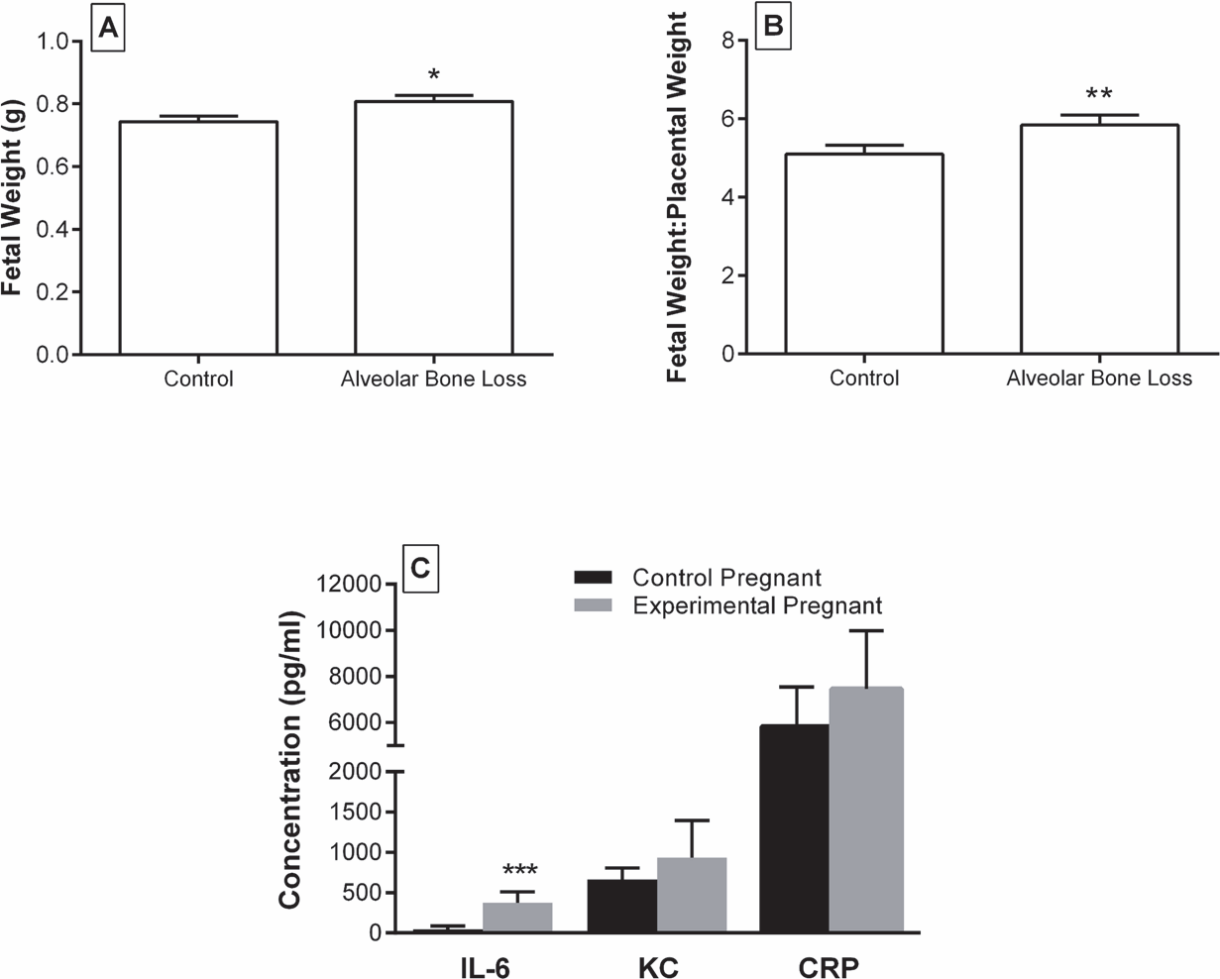
Birth outcomes and IL-6, KC and CRP levels in pregnant mice following experimentally induced periodontitis. (A) Mean fetal weight and (B) Mean fetal weight: placental weight ratio at day 18 of gestation in mice with alveolar bone loss compared to no alveolar bone loss (*p = 0.015, ** p = 0.030) and (C) circulating concentration of IL-6, KC in serum of pregnant mice with induced periodontitis compared to controls (***p = 0.047). Bars represent the mean ± SEM.

After adjusting for litter size and experimental or control group replicates, the mean fetal weight at day 18 of gestation was significantly (p = 0.015) higher in mice with alveolar bone loss (0.808 ± 0.019g) compared to controls (0.744 ± 0.017 g, Fig. 4A). Mean placental weights were similar between mice with and without alveolar bone loss. However, fetal weight:placental weight ratio was 14.6% higher (p = 0.030) in mice with alveolar bone loss (5.839 ± 0.253) compared with controls (5.094 ± 0.227, Fig. 4B). Mean litter size, numbers of viable fetuses and resorptions and mean maternal weight at day 18 of gestation were similar between mice and controls.

Circulating levels of IL-6 were increased by 28% (p = 0.047) in pregnant control mice (Fig. 4C). In contrast, serum levels of KC (mouse homolog of IL-8) and CRP were not significantly different between mice with experimentally induced periodontitis and controls.

## Discussion

The hypothesis that periodontal disease may stimulate APO has been investigated for over 20 years [51]. Although there is not yet enough evidence to say unequivocally that periodontitis can cause adverse pregnancy outcomes, data supporting this relationship continue to increase [2,19,30,52]. Although the causality of APO’s are multifactorial, the predominant view has been that the placental and fetal membranes are essentially sterile and that infection ascends from the lower genital tract. However, more recently it has been proposed that the placenta has its own microbiome and that colonisation and infection can occur by the haematogenous migration of bacteria from predominantly oral tissues [4,20].

The primary role of *F*. *nucleatum* in the aetiology of periodontal disease is to provide a sub-gingival habitat which promotes the proliferation and colonisation of more aggressively virulent organisms such as *P*. *gingivalis*. The ability to form a biofilm when under environmental stress, survive in the oxygen rich circulation and proliferate in a new environment has been a hallmark of the species and may be central to its ability to transmigrate haematogenously to other organs in the body [26,53,54]. The level of genetic heterogeneity among the species is comparatively large and may have resulted from a high level of horizontal gene transfer occurring within the densely populated confines of the sub-gingival milieu [44,46,53]. Its ability to aggregate with other bacteria and invade the endothelium also implies that *F*. *nucleatum* may also be responsible for the haematogenous spread of other Gram negative anaerobes such as *P*. *gingivalis* and *Capnocytophaga* which have also been found in neonatal gastric aspirates but not in the maternal vagina [29,55].

The use of an experimental animal model to assess the relationship between periodontal disease and APO’s overcomes some of the limitations associated with studies using human subjects as factors such as the duration of the disease and susceptibility are easier to control [56]. The oral gavage model used in the present study produced significant alveolar bone loss which has been previously shown in BALB/c mice given a dual inoculum of *P*. *gingivalis* and *F*. *nucleatum* [57]. We also used published protocols [19,27] to introduce a transient bacteraemia of *F*. *nucleatum* directly into the circulatory system of mice to determine whether the ability for haematogenous transit, infection of the placenta and APO was subspecies specific. It is thought that bacteraemia occurs with periodontal disease (which is predominantly a chronic infection) as a result of gingival bleeding [4]. The absence of a significant difference between maternal weights in each experimental group, or any systemic signs indicates that our findings are not likely to be a result of systemic infection by *F*. *nucleatum*. *FNN* was detected in the placenta by PCR 48 hours after tail vein injection and therefore was able to colonise it presumably facilitated by its immuno-tolerant state. *FNN* was also the only subspecies that caused significant fetal miscarriage (resorption) in late gestation (day 16 equivalent to third trimester). Han *et al*. (2004) found that *F*. *nucleatum* established stable colonisation in the placentas of mice and proliferated rapidly over time [27]. By 48–72 hours post-injection, *F*. *nucleatum* had spread to the amniotic fluid and fetuses which coincided with observed fetal death. In the present study, 48 hours after injection with *FNN*, mice were sacrificed and fetal death was noted in 40% of fetuses. Han *et al*. (2004) also reported that placental infection was dose dependent and was proportional to fetal death [27]. It has previously been reported that this ability may be strain specific as Han *et al*., (2000) reported that strain 12230 (a clinical transtracheal isolate) *FNN* and *FNP* were highly invasive and adhered strongly to KB cells while other strains were not [36].

Although no evidence was found that *FNP*, *FNV* and *FNF* could migrate and infect the placenta, injection of *FNP*, *FNV* and *FNN* resulted in significant APO which may have been due to a systemic inflammatory reaction in response to the I.V. inoculation of bacteria. To test whether the observed APO were due to the release of major components of the cell wall such as LPS, we used heat killed *FNN* as an additional control group and found that the viable bacteria were required as no APO were observed in these mice.

Inflammatory mediators/cytokines play a pivotal role in APO. Pregnancy is an immuno-tolerant state which is induced to accept foreign paternal antigens so that the conceptus is not rejected. Given the close relationship between inflammation and infection, it seems likely that alterations to the levels of inflammatory mediators and cytokines resulting from a normal host response to an infectious agent may cause an imbalance in cytokine levels. Therefore, if the inflammatory response reaches a certain threshold caused by the presence and abundance of bacteria, negative pregnancy outcomes may occur. Indeed a significant percentage of PTB, particularly very early PTB, in humans is associated with infection [4].

Following I.V. injection, CRP levels were significantly higher in mice injected with *FNN* compared to controls and non-pregnant females which may have been due to an increased inflammatory response to infection by *FNN* (Fig. 2D). This acute phase protein is a general marker for systemic inflammation and increases in response to the release of pro-inflammatory cytokines. It has previously been associated with PTB, IUGR and preeclampsia [4,58]. However in the present study, PTB was not one of the APO associated with I.V. injection of *FNN*. It should also be noted that I.V. injection of *FNP*, *FNF* and *FNV* did not produce significant changes to serum CRP levels. However 3 mice were used from each group which may have been insufficient to determine significance.

IL-1β and IL-6 have been identified as the key inflammatory mediators in periodontal disease which lead to connective tissue degradation and alveolar bone resorption [59,60]. Circulating TNF-α and IL-6 are also thought to become elevated as a result of intrauterine infections in humans, [61] however our results showed that only IL-6 was significantly increased in mice with induced periodontitis. This effect has been demonstrated previously using a Baboon model of ligature induced periodontitis, whereby IL-6 levels were found to be directly proportional to severity of periodontitis [62]. Increased levels of IL-6 are thought to be more destructive to the fetus earlier in gestation as animal studies have shown that the placental barrier is more permeable mid gestation [63].

One of the factors influencing the outcome and therefore the level of APO is the numbers of bacteria entering the circulation. The degree of bacteraemia has been shown to be related to the gingival index, plaque index and the number of sites with bleeding on probing [64]. The prevalence of *F*. *nucleatum* in early gingivitis through to severe periodontitis and its ability to invade the endothelium implies that the organism can gain access to the circulation irrespective of the severity of periodontal disease [4,64]. This may explain why intervention studies where periodontal therapy has been provided during pregnancy has not reduced the incidence of APO [4]. We suggest that treatment prior to pregnancy would be more efficacious.

Given the delicate balance during pregnancy with a relatively immuno-suppressed state, the magnitude of disturbances to the inflammatory response in the feto-placental unit is a significant factor in APO. If the disruption of the inflammatory response is mild, pregnancy outcomes may be less severe. Interestingly, we found that direct inoculation of *FNF* or mice with induced periodontitis produced significantly larger pups. An increase in fetal weight is a pregnancy outcome most frequently associated with maternal glucose intolerance such as in gestational diabetes and uncontrolled diabetes mellitus [65–67]. The complications of fetal macrosomia include trauma to the baby and mother during birth, fetal death and decreased Apgar scores [68]. Other adverse health effects associated with glucose intolerance during pregnancy include preeclampsia and PTB, which have been reported in association with maternal periodontitis [69]. In normal pregnancy the mother becomes relatively insulin resistant permitting the transport of sufficient glucose from the maternal to the fetal circulations via facilitated diffusion along a concentration gradient. Although fasting blood glucose measurements were not taken, we hypothesise that mice with periodontitis may have had elevated blood glucose concentrations that would enhance fetal growth possibly due to sub-clinical systemic inflammation associated with periodontitis. An increase in fetal weight:placental weight ratio also occurs in some cases of pregnancy-induced hypertension, although this is most notably due to a reduced size of the placenta as opposed to increased growth of the fetus. In such cases, it is hypothesised that cytokines are released into the maternal circulation in order to maximise placental function by increasing maternal blood pressure [70]. This would increase placental perfusion with maternal blood and facilitate greater nutrient transport to the fetus. These responses may be enhanced by periodontitis producing further systemic inflammatory responses and potentially insulin resistance. This phenomenon may be associated with higher blood pressure, increasing perfusion of the placenta and enhancing fetal growth. A causal role of periodontitis in gestational diabetes and diabetes mellitus has been investigated in a number of studies although results so far have not been conclusive [71]. Interestingly it has previously been reported that women with gestational diabetes mellitus show elevated serum levels of CRP, IL-6 and TNF-α. Thus prolonged elevated levels of IL-6 and TNF-α can interfere with carbohydrate metabolism which can lead to glucose intolerance and diabetes mellitus [4]. In mice with induced periodontitis we observed significantly increased levels of IL-6 which may be consistent with the observed increase in fetal weight.

It has also been reported that the timing of the microbial challenge can also lead to the activation of different inflammatory pathways by the same pathogen, and this may explain the differences in APO seen in this and other animal studies [63]. In the present study, the induction of periodontitis over a longer time frame compared to other studies and before pregnancy may more closely reflect the situation in human populations.

Our findings have identified further areas of study and have contributed towards identifying a causal mechanism between periodontitis and APO. There is an abundance of evidence for the safe treatment of periodontal disease before and during pregnancy, enabling an improved oral environment to reduce infection and inflammation that may act through the mother to harm the unborn child. Improved communication between mothers, obstetricians and dental professionals may be an effective early strategy to reduce the risk of periodontitis and perhaps APO.

## References

[1] Amano A (2010) Host-parasite interactions in periodontitis: subgingival infection and host sensing. Periodontol 2000 52: 7–11. 10.1111/j.1600-0757.2009.00328.x 20017792

[2] Han YW, Wang X (2013) Mobile microbiome: oral bacteria in extra-oral infections and inflammation. J Dent Res 92: 485–491. 10.1177/0022034513487559 23625375 PMC3654760

[3] Offenbacher S, Katz V, Fertik G, Collins J, Boyd D, Maynor G, et al (1996) Periodontal infection as a possible risk factor for preterm low birth weight. J Periodontol 67: 1103–1113. 8910829 10.1902/jop.1996.67.10s.1103

[4] Madianos PN, Bobetsis YA, Offenbacher S (2013) Adverse pregnancy outcomes (APOs) and periodontal disease: pathogenic mechanisms. J Clin Periodontol 40 Suppl 14: S170–180. 10.1111/jcpe.12082 23627327

[5] Jeffcoat M, Parry S, Sammel M, Clothier B, Catlin A, Macones G (2010) Periodontal infection and preterm birth: successful periodontal therapy reduces the risk of preterm birth. BJOG 118: 250–256. 10.1111/j.1471-0528.2010.02713.x 20840689

[6] Jeffcoat MK, Geurs NC, Reddy MS, Cliver SP, Goldenberg RL, Hauth JC (2001) Periodontal infection and preterm birth: results of a prospective study. J Am Dent Assoc 132: 875–880. 11480640 10.14219/jada.archive.2001.0299

[7] Lopez NJ, Smith PC, Gutierrez J (2002) Periodontal therapy may reduce the risk of preterm low birth weight in women with periodontal disease: a randomized controlled trial. J Periodontol 73: 911–924. 12211502 10.1902/jop.2002.73.8.911

[8] Lopez NJ, Smith PC, Gutierrez J (2002) Higher risk of preterm birth and low birth weight in women with periodontal disease. J Dent Res 81: 58–63. 11820369 10.1177/002203450208100113

[9] Macones GA, Parry S, Nelson DB, Strauss JF, Ludmir J, Cohen AW, et al (2010) Treatment of localized periodontal disease in pregnancy does not reduce the occurrence of preterm birth: results from the Periodontal Infections and Prematurity Study (PIPS). Am J Obstet Gynecol 202: 147 e141–148. 10.1016/j.ajog.2009.10.892 20113691

[10] Michalowicz BS, Hodges JS, DiAngelis AJ, Lupo VR, Novak MJ, Ferguson JE, et al (2006) Treatment of periodontal disease and the risk of preterm birth. N Engl J Med 355: 1885–1894. 17079762 10.1056/NEJMoa062249

[11] Newnham JP, Newnham IA, Ball CM, Wright M, Pennell CE, Swain J, et al (2009) Treatment of periodontal disease during pregnancy: a randomized controlled trial. Obstet Gynecol 114: 1239–1248. 10.1097/AOG.0b013e3181c15b40 19935025

[12] Offenbacher S, Lin D, Strauss R, McKaig R, Irving J, Barros SP, et al (2006) Effects of periodontal therapy during pregnancy on periodontal status, biologic parameters, and pregnancy outcomes: a pilot study. J Periodontol 77: 2011–2024. 17209786 10.1902/jop.2006.060047

[13] Oliveira AM, de Oliveira PA, Cota LO, Magalhaes CS, Moreira AN, Costa FO (2011) Periodontal therapy and risk for adverse pregnancy outcomes. Clin Oral Investig 15: 609–615. 10.1007/s00784-010-0424-8 20495936

[14] Tarannum F, Faizuddin M (2007) Effect of periodontal therapy on pregnancy outcome in women affected by periodontitis. J Periodontol 78: 2095–2103. 17970675 10.1902/jop.2007.060388

[15] Australian Research Centre for Population Oral Health TUoASA (2009) Periodontal diseases in the Australian adult population. Aust Dent J 54: 390–393. 10.1111/j.1834-7819.2009.01167.x 20415940

[16] Eke PI, Dye BA, Wei L, Thornton-Evans GO, Genco RJ, Beck J, et al (2012) Prevalence of periodontitis in adults in the United States: 2009 and 2010. J Dent Res 91: 914–920. 22935673 10.1177/0022034512457373

[17] Beck S, Wojdyla D, Say L, Betran AP, Merialdi M, Requejo JH, et al (2010) The worldwide incidence of preterm birth: a systematic review of maternal mortality and morbidity. Bull World Health Organ 88: 31–38. 10.2471/BLT.08.062554 20428351 PMC2802437

[18] Behrman R, Butler A (2007) Committee on Understanding Premature Birth and Assuring Healthy Outcomes Preterm Birth: Causes, Consequences, and Prevention. Washington, D.C: The National Academies Press. 20669423

[19] Fardini Y, Chung P, Dumm R, Joshi N, Han YW (2010) Transmission of diverse oral bacteria to murine placenta: evidence for the oral microbiome as a potential source of intrauterine infection. Infect Immun 78: 1789–1796. 10.1128/IAI.01395-09 20123706 PMC2849412

[20] Prince AL, Antony KM, Ma J, Aagaard KM (2014) The microbiome and development: a mother's perspective. Semin Reprod Med 32: 14–22. 10.1055/s-0033-1361818 24390916

[21] Bobetsis YA, Barros SP, Offenbacher S (2006) Exploring the relationship between periodontal disease and pregnancy complications. J Am Dent Assoc 137 Suppl: 7S–13S. 17012730 10.14219/jada.archive.2006.0403

[22] Africa CW (2011) Oral colonization of Gram-negative anaerobes as a risk factor for preterm delivery. Virulence 2: 498–508. 10.4161/viru.2.6.17719 21971188

[23] Han YW (2011) Oral health and adverse pregnancy outcomes—what's next? J Dent Res 90: 289–293. 10.1177/0022034510381905 21041548 PMC3144105

[24] Socransky SS, Haffajee AD, Cugini MA, Smith C, Kent RL Jr. (1998) Microbial complexes in subgingival plaque. J Clin Periodontol 25: 134–144. 9495612 10.1111/j.1600-051x.1998.tb02419.x

[25] Arce RM, Barros SP, Wacker B, Peters B, Moss K, Offenbacher S (2009) Increased TLR4 expression in murine placentas after oral infection with periodontal pathogens. Placenta 30: 156–162. 10.1016/j.placenta.2008.11.017 19101032 PMC2656361

[26] Diaz P, Zilm P, Rogers A (2002) Fusobacterium nucleatum supports the growth of Porphyromonas gingivalis in oxygenated and carbon dioxide-depleted environments. Microbiology 148: 467–472. 11832510 10.1099/00221287-148-2-467

[27] Han YW, Redline RW, Li M, Yin L, Hill GB, McCormick TS (2004) Fusobacterium nucleatum induces premature and term stillbirths in pregnant mice: implication of oral bacteria in preterm birth. Infect Immun 72: 2272–2279. 15039352 10.1128/IAI.72.4.2272-2279.2004 PMC375172

[28] Kolenbrander PE, Andersen RN, Moore LV (1989) Coaggregation of Fusobacterium nucleatum, Selenomonas flueggei, Selenomonas infelix, Selenomonas noxia, and Selenomonas sputigena with strains from 11 genera of oral bacteria. Infect Immun 57: 3194–3203. 2777378 10.1128/iai.57.10.3194-3203.1989 PMC260789

[29] Gonzales-Marin C, Spratt DA, Allaker R (2012) Maternal oral origin of Fusobacterium nucleatum in adverse pregnancy outcomes as determined using the 16S-23S rDNA intergenic transcribed spacer region. J Med Microbiol. 10.1099/jmm.0.049452-0 23002071

[30] Han YW, Fardini Y, Chen C, Iacampo KG, Peraino VA, Shamonki JM, et al (2010) Term stillbirth caused by oral Fusobacterium nucleatum. Obstet Gynecol 115: 442–445. 10.1097/AOG.0b013e3181cb9955 20093874 PMC3004155

[31] Wang X, Buhimschi CS, Temoin S, Bhandari V, Han YW, Buhimschi IA (2013) Comparative microbial analysis of paired amniotic fluid and cord blood from pregnancies complicated by preterm birth and early-onset neonatal sepsis. PLoS One 8: e56131 10.1371/journal.pone.0056131 23437088 PMC3577789

[32] Castellarin M, Warren RL, Freeman JD, Dreolini L, Krzywinski M, Strauss J, et al (2012) Fusobacterium nucleatum infection is prevalent in human colorectal carcinoma. Genome Res 22: 299–306. 10.1101/gr.126516.111 22009989 PMC3266037

[33] Kostic AD, Gevers D, Pedamallu CS, Michaud M, Duke F, Earl AM, et al (2012) Genomic analysis identifies association of Fusobacterium with colorectal carcinoma. Genome Res 22: 292–298. 10.1101/gr.126573.111 22009990 PMC3266036

[34] Strauss J, Kaplan GG, Beck PL, Rioux K, Panaccione R, Devinney R, et al (2011) Invasive potential of gut mucosa-derived Fusobacterium nucleatum positively correlates with IBD status of the host. Inflamm Bowel Dis 17: 1971–1978. 10.1002/ibd.21606 21830275

[35] Temoin S, Chakaki A, Askari A, El-Halaby A, Fitzgerald S, Marcus RE, et al (2012) Identification of oral bacterial DNA in synovial fluid of patients with arthritis with native and failed prosthetic joints. J Clin Rheumatol 18: 117–121. 10.1097/RHU.0b013e3182500c95 22426587 PMC3888235

[36] Han YW, Shi W, Huang GT, Kinder Haake S, Park NH, Kuramitsu H, et al (2000) Interactions between periodontal bacteria and human oral epithelial cells: Fusobacterium nucleatum adheres to and invades epithelial cells. Infect Immun 68: 3140–3146. 10816455 10.1128/iai.68.6.3140-3146.2000 PMC97547

[37] Hill GB (1998) Preterm birth: associations with genital and possibly oral microflora. Ann Periodontol 3: 222–232. 9722706 10.1902/annals.1998.3.1.222

[38] Rogers AH, Zilm PS, Gully NJ, Pfennig AL, Marsh PD (1991) Aspects of the growth and metabolism of Fusobacterium nucleatum ATCC 10953 in continuous culture. Oral Microbiol Immunol 6: 250–255. 1812468 10.1111/j.1399-302x.1991.tb00486.x

[39] Xu M, Yamada M, Li M, Liu H, Chen SG, Han YW (2007) FadA from Fusobacterium nucleatum utilizes both secreted and nonsecreted forms for functional oligomerization for attachment and invasion of host cells. J Biol Chem 282: 25000–25009. 17588948 10.1074/jbc.M611567200

[40] Liu H, Redline RW, Han YW (2007) Fusobacterium nucleatum induces fetal death in mice via stimulation of TLR4-mediated placental inflammatory response. J Immunol 179: 2501–2508. 17675512 10.4049/jimmunol.179.4.2501

[41] Dzink JL, Sheenan MT, Socransky SS (1990) Proposal of three subspecies of Fusobacterium nucleatum Knorr 1922: Fusobacterium nucleatum subsp. nucleatum subsp. nov., comb. nov.; Fusobacterium nucleatum subsp. polymorphum subsp. nov., nom. rev., comb. nov.; and Fusobacterium nucleatum subsp. vincentii subsp. nov., nom. rev., comb. nov. Int J Syst Bacteriol 40: 74–78. 2223601 10.1099/00207713-40-1-74

[42] Gharbia SE, Shah HN (1992) Fusobacterium nucleatum subsp. fusiforme subsp. nov. and Fusobacterium nucleatum subsp. animalis subsp. nov. as additional subspecies within Fusobacterium nucleatum. Int J Syst Bacteriol 42: 296–298. 1581188 10.1099/00207713-42-2-296

[43] Gharbia SE, Shah HN, Lawson PA, Haapasalo M (1990) Distribution and frequency of Fusobacterium nucleatum subspecies in the human oral cavity. Oral Microbiol Immunol 5: 324–327. 2098710 10.1111/j.1399-302x.1990.tb00434.x

[44] Kapatral V, Anderson I, Ivanova N, Reznik G, Los T, Lykidis A, et al (2002) Genome sequence and analysis of the oral bacterium Fusobacterium nucleatum strain ATCC 25586. J Bacteriol 184: 2005–2018. 11889109 10.1128/JB.184.7.2005-2018.2002 PMC134920

[45] Kapatral V, Ivanova N, Anderson I, Reznik G, Bhattacharyya A, Gardner WL, et al (2003) Genome analysis of F. nucleatum sub spp vincentii and its comparison with the genome of F. nucleatum ATCC 25586. Genome Res 13: 1180–1189. 12799352 10.1101/gr.566003 PMC403646

[46] Karpathy SE, Qin X, Gioia J, Jiang H, Liu Y, Liu Y, et al (2007) Genome sequence of Fusobacterium nucleatum subspecies polymorphum—a genetically tractable fusobacterium. PLoS ONE 2: e659 17668047 10.1371/journal.pone.0000659 PMC1924603

[47] Yeo A, Smith MA, Lin D, Riche EL, Moore A, Elter J,. et al (2005) Campylobacter rectus mediates growth restriction in pregnant mice. J Periodontol 76: 551–557. 15857095 10.1902/jop.2005.76.4.551

[48] Baker PJ, Dixon M, Roopenian DC (2000) Genetic control of susceptibility to Porphyromonas gingivalis-induced alveolar bone loss in mice. Infect Immun 68: 5864–5868. 10992496 10.1128/iai.68.10.5864-5868.2000 PMC101548

[49] Bendyk A, Marino V, Zilm PS, Howe P, Bartold PM (2009) Effect of dietary omega-3 polyunsaturated fatty acids on experimental periodontitis in the mouse. J Periodontal Res 44: 211–216. 10.1111/j.1600-0765.2008.01108.x 19210341

[50] Glasel JA (1995) Validity of nucleic acid purities monitored by 260nm/280nm absorbance ratios. Biotechniques 18: 62–63. 7702855

[51] Collins JG, Windley HW 3rd, Arnold RR, Offenbacher S (1994) Effects of a Porphyromonas gingivalis infection on inflammatory mediator response and pregnancy outcome in hamsters. Infect Immun 62: 4356–4361. 7927695 10.1128/iai.62.10.4356-4361.1994 PMC303116

[52] Liu P, Liu Y, Wang J, Guo Y, Zhang Y, Xiao S. (2014) Detection of fusobacterium nucleatum and fadA adhesin gene in patients with orthodontic gingivitis and non-orthodontic periodontal inflammation. PLoS One 9: e85280 10.1371/journal.pone.0085280 24416378 PMC3887018

[53] Zilm PS, Mira A, Bagley CJ, Rogers AH (2010) Effect of alkaline growth pH on the expression of cell envelope proteins in Fusobacterium nucleatum. Microbiology 156: 1783–1794. 10.1099/mic.0.035881-0 20299401

[54] Zilm PS, Rogers AH (2007) Co-adhesion and biofilm formation by Fusobacterium nucleatum in response to growth pH. Anaerobe 13: 146–152. 17540586 10.1016/j.anaerobe.2007.04.005

[55] Carta G, Persia G, Falciglia K, Iovenitti P (2004) Periodontal disease and poor obstetrical outcome. Clin Exp Obstet Gynecol 31: 47–49. 14998188

[56] de Molon RS, de Avila ED, Boas Nogueira AV, Chaves de Souza JA, Avila-Campos MJ, de Andrade CR, et al (2014) Evaluation of the host response in various models of induced periodontal disease in mice. J Periodontol 85: 465–477. 10.1902/jop.2013.130225 23805811

[57] Polak D, Wilensky A, Shapira L, Halabi A, Goldstein D, Weiss EI, et al (2009) Mouse model of experimental periodontitis induced by Porphyromonas gingivalis/Fusobacterium nucleatum infection: bone loss and host response. J Clin Periodontol 36: 406–410. 10.1111/j.1600-051X.2009.01393.x 19419440

[58] Pitiphat W, Gillman MW, Joshipura KJ, Williams PL, Douglass CW, Rich-Edwards JW (2005) Plasma C-reactive protein in early pregnancy and preterm delivery. Am J Epidemiol 162: 1108–1113. 16236995 10.1093/aje/kwi323 PMC1994922

[59] Cochran DL (2008) Inflammation and bone loss in periodontal disease. J Periodontol 79: 1569–1576. 10.1902/jop.2008.080233 18673012

[60] Graves DT, Cochran D (2003) The contribution of interleukin-1 and tumor necrosis factor to periodontal tissue destruction. J Periodontol 74: 391–401. 12710761 10.1902/jop.2003.74.3.391

[61] Keelan JA, Wong PM, Bird PS, Mitchell MD (2010) Innate inflammatory responses of human decidual cells to periodontopathic bacteria. Am J Obstet Gynecol 202: 471 e471–411. 10.1016/j.ajog.2010.02.031 20452492

[62] Ebersole JL, Steffen MJ, Holt SC, Kesavalu L, Chu L, Cappelli D (2010) Systemic inflammatory responses in progressing periodontitis during pregnancy in a baboon model. Clin Exp Immunol 162: 550–559. 10.1111/j.1365-2249.2010.04202.x 21070210 PMC3026559

[63] Michelin MC, Teixeira SR, Ando-Suguimoto ES, Lucas SR, Mayer MP (2012) Porphyromonas gingivalis infection at different gestation periods on fetus development and cytokines profile. Oral Dis 18: 648–654. 10.1111/j.1601-0825.2012.01917.x 22471815

[64] Forner L, Larsen T, Kilian M, Holmstrup P (2006) Incidence of bacteremia after chewing, tooth brushing and scaling in individuals with periodontal inflammation. J Clin Periodontol 33: 401–407. 16677328 10.1111/j.1600-051X.2006.00924.x

[65] Casey BM, Lucas MJ, McIntire DD, Leveno KJ (1997) Pregnancy outcomes in women with gestational diabetes compared with the general obstetric population. Obstet Gynecol 90: 869–873. 9397092 10.1016/s0029-7844(97)00542-5

[66] Kwik M, Seeho SK, Smith C, McElduff A, Morris JM (2007) Outcomes of pregnancies affected by impaired glucose tolerance. Diabetes Res Clin Pract 77: 263–268. 17275121 10.1016/j.diabres.2006.12.004

[67] Landon MB, Spong CY, Thom E, Carpenter MW, Ramin SM, Casey B, et al (2009) A multicenter, randomized trial of treatment for mild gestational diabetes. N Engl J Med 361: 1339–1348. 10.1056/NEJMoa0902430 19797280 PMC2804874

[68] Spellacy WN, Miller S, Winegar A, Peterson PQ (1985) Macrosomia—maternal characteristics and infant complications. Obstet Gynecol 66: 158–161. 4022478

[69] Xiong X, Saunders LD, Wang FL, Demianczuk NN (2001) Gestational diabetes mellitus: prevalence, risk factors, maternal and infant outcomes. Int J Gynaecol Obstet 75: 221–228. 11728481 10.1016/s0020-7292(01)00496-9

[70] Imada S, Takagi K, Kikuchi A, Ishikawa K, Tamaru S, Horikoshi T, et al (2012) Birthweight placental weight ratio of appropriate-for-dates and light-for-dates infants in preterm delivery. J Obstet Gynaecol Res 38: 122–129. 10.1111/j.1447-0756.2011.01641.x 21917076

[71] Chang PC, Lim LP (2012) Interrelationships of periodontitis and diabetes: A review of the current literature. Journal of Dental Sciences 7: 272–282.

